# Role of Weekday Variation on Glucose, Insulin, and Triglyceride: A Cross-Sectional Analysis From the Maastricht Study

**DOI:** 10.1210/clinem/dgac286

**Published:** 2022-05-16

**Authors:** Kim K B Clemmensen, Annemarie Koster, Yannick T H Nielen, Pieter C Dagnelie, Coen D A Stehouwer, Hans Bosma, Anke Wesselius, Kristine Færch, Simone J P M Eussen

**Affiliations:** Steno Diabetes Center Copenhagen, Gentofte, Denmark; Department of Social Medicine, Maastricht University, Maastricht, The Netherlands; School for Care and Public Health Research Institute (CAPHRI), Maastricht University, Maastricht, The Netherlands; Department of Clinical Pharmacy and Toxicology, Maastricht University Medical Center+, Maastricht, The Netherlands; School for Cardiovascular Diseases (CARIM), Maastricht University, Maastricht, The Netherlands; School for Cardiovascular Diseases (CARIM), Maastricht University, Maastricht, The Netherlands; Department of Internal Medicine, Maastricht University Medical Centre, Maastricht, The Netherlands; Department of Social Medicine, Maastricht University, Maastricht, The Netherlands; School for Care and Public Health Research Institute (CAPHRI), Maastricht University, Maastricht, The Netherlands; Department of Complex Genetics and Epidemiology, School of Nutrition and Translational Research in Metabolism, Maastricht University, Maastricht, The Netherlands; Steno Diabetes Center Copenhagen, Gentofte, Denmark; Department of Biomedical Sciences, University of Copenhagen, Copenhagen, Denmark; School for Care and Public Health Research Institute (CAPHRI), Maastricht University, Maastricht, The Netherlands; School for Cardiovascular Diseases (CARIM), Maastricht University, Maastricht, The Netherlands; Department of Epidemiology, Maastricht University, Maastricht, The Netherlands

**Keywords:** metabolism, triglyceride, weekday, oral glucose tolerance test

## Abstract

**Context:**

The timing of sleep, physical activity, and dietary intake show variation over the week, with different timings in the weekend compared to the weekdays, which may potentially lead to impaired glucose and lipid regulation on Mondays compared to other weekdays.

**Objective:**

The aim of the study was to investigate differences in glucose metabolism and fasting triglyceride concentrations on Mondays compared to the rest of the week.

**Design, setting and participants:**

This cross-sectional study is based on data from the Maastricht Study, including 6067 participants without known diabetes and 1568 previously diagnosed with type 2 diabetes.

**Main outcome measures:**

Confounder-adjusted linear regression analysis was applied to study the associations of day of the week of examination with glucose and insulin responses to an oral glucose tolerance test and fasting triglyceride concentrations.

**Results:**

In fully confounder-adjusted models, mean (95% CI) concentrations of fasting glucose, insulin, and triglycerides were slightly higher on Mondays compared with the other weekdays [glucose: 1% (0-2); insulin: 9% (1-18); triglycerides: 5% (2-8)]. Interaction analyses revealed that the association of weekday with insulin was only pronounced in men [18% (3-35)], but not in women [1% (−8-10)], whereas the associations with glucose and triglycerides were only apparent for individuals with known type 2 diabetes [glucose: 4% (0-7); triglycerides: 14% (6-23)] compared to the background population [glucose: 0% (0-1); triglycerides: 3% (0-6)].

**Discussion:**

Being examined on a Monday was associated with higher fasting insulin concentrations among men but not women.

Cardiometabolic disorders constitute a burden on health and economy both for the individual and society (1). It is well-established that lifestyle factors such as sleep, physical activity, and diet are associated with the risk of cardiometabolic disorders (2, 3). Circadian timing of sleep, physical activity, and dietary intake differ during the week, with different timings in the weekend compared to the weekdays (4-6). Irregularity between days due to social activities is termed *social jetlag* (7, 8). A study on social jetlag found that the sleep midpoint differed by more than 1 hour in the weekend compared to weekdays for almost 40% of the study population (6). Furthermore, a study based on the National Health and Nutrition Examination Survey found a higher energy intake as well as a poorer diet quality on weekends compared to weekdays (5). This pattern was also found in a recent Danish study (4). These variations in behavior over the week might translate into variation in cardiometabolic risk factors during the weekdays. This is supported by studies examining variations in markers of glucose and lipid metabolism during the week. In a study of 807 children insulin resistance (homeostatic model assessment for insulin resistance) was 30% higher on Mondays than on Fridays (9). A recent study from our group investigated the associations of weekday of examination with glucose and insulin concentrations during an oral glucose tolerance test (OGTT) (10). We found similar fasting glucose concentrations but higher fasting insulin on Mondays compared to the other weekdays, suggesting a worse glucose regulation following the weekend (10).

Several studies have observed lower triglyceride concentrations on Fridays compared to Mondays with differences of 5% to 28% (9, 11, 12). Taken together this points to an adverse effect of weekend behavior on triglyceride concentrations. These studies were performed in children (9) or adolescents (12) or on samples collected as part of routine care (11); it would therefore be interesting to examine this in an adult population sampled from the general population.

The objective of this study was to examine whether the day of the week is associated with concentrations of plasma glucose, plasma insulin, and serum triglycerides in the fasted state and glucose and insulin concentrations during an OGTT. We hypothesized that fasting glucose, insulin, and triglyceride concentrations are higher and that the metabolic response to an OGTT is worse on Mondays compared to the other days of the week.

## Materials and Methods

The study is based on data from the Maastricht Study, an observational prospective population-based cohort study. The rationale and methodology have been described previously (13). In brief, The Maastricht Study is an extensive phenotyping study with a focus on type 2 diabetes and its etiology, pathophysiology, and comorbidities. Individuals aged 45 to 70 years living in the southern part of the Netherlands were eligible for inclusion. Participants were recruited through mass media campaigns and from the municipal registries and the regional Diabetes Patient Registry via mailings. The study population had an oversampling of individuals with known type 2 diabetes (13), for reasons of efficiency. The present study includes cross-sectional data on the first 7689 individuals who completed the baseline survey between November 2010 and December 2017. The examinations of each participant were performed within a time window of 3 months. For the present analysis, 50 individuals with other types of diabetes than type 2 diabetes were excluded. Furthermore, 4 participants with no fasting blood glucose or only a finger-prick fasting glucose measurement were excluded, leaving 7635 for analyses.

The Maastricht Study has been approved by the institutional medical ethical committee (NL31329.068.10) and the Ministry of Health, Welfare and Sports of the Netherlands (131088-105234-PG). All participants gave written informed consent.

### Measurements

All measurements were performed by trained research staff at the Maastricht Study research center following standardized protocols. The fasting samples and OGTTs were performed on Mondays to Saturdays, with samples from 1443 participants on Mondays, 1568 on Tuesdays, 1601 on Wednesdays, 1445 on Thursdays, 1330 on Fridays, and 248 on Saturdays. Blood sampling was scheduled according to participants’ preference and availability. We dichotomized the weekday variable into Mondays vs the other days of the week combined to ease interpretation and to utilize the full data set. Also, we believe that the effect of weekend behavior is best seen on Mondays as it follows a full weekend.

### Outcomes

A standard 75-g OGTT was performed after an overnight fast, with blood samples at baseline and 15, 30, 45, 60, 90, and 120 minutes after glucose ingestion; not all individuals have full data from the OGTT. Participants were instructed not to eat and drink, except water, after 11 pm the evening prior to blood collection. Individuals with a fasting glucose concentration >11.0 mmol/L measured with a finger prick and individuals on insulin treatment did not undergo an OGTT for safety reasons (13). Fasting and 120-minute postload plasma glucose were measured by the enzymatic hexokinase method, and fasting triglyceride concentrations were measured by the enzymatic colorimetric method, by Roche Cobas systems (Roche Diagnostics, Mannheim, Germany). Fasting and 120-minute postload plasma insulin concentrations were measured by a custom duplex array of MesoScale Discovery Platform (MSD, Gaithersburg, MD, USA; RRID: AB_2819057, https://scicrunch.org/resolver/AB_2819057).

### Calculations

Early and total responses to the OGTT for plasma glucose and insulin concentrations were calculated as total areas under curves (tAUCs) using the trapezoid rule. Relative early response (rAUC_0-30min_) was calculated as the ratio tAUC_0-30min_/(fasting concentration × 30 min) and relative total response (rAUC_0-120min_) as tAUC_0-120min_/(fasting concentration × 120 min). The rAUC reflects the change in the concentrations relative to the fasting concentration. An advantage of calculating the rAUC instead of the incremental AUC is that rAUC is always positive and can therefore be logarithmically transformed if needed.

### Covariates

Diabetes status was classified according to the World Health Organization 2006 criteria (14). Blood pressure (mmHg), weight, and height were measured at the study visits using standardized procedures. Information on education, physical activity, smoking, energy intake, retirement status, and family history of cardiovascular disease and diabetes was self-reported. Physical activity was assessed both by questionnaires and measured by accelerometers [activ-PAL3 physical activity monitor (PAL Technologies, Glasgow, UK)] for 8 days. The difference between weekend and weekdays in time spent in bed (15) and time spent in moderate-to-vigorous physical activity from accelerometers were assessed by subtracting the minutes used on weekdays from time used on weekend days. Time spent in bed from Friday and Saturday nights to Saturday and Saturday morning, respectively, was defined as weekend nights whereas physical activity was defined from morning to evening Saturday and Sunday, respectively. Adherence to the Dutch Healthy Diet Index (16) was assessed and based on a validated food frequency questionnaire (17).

### Statistical Analyses

Descriptive statistics are presented for the total population and stratified by weekday of blood sampling (Monday vs Tuesday-Saturday). Differences in characteristics between weekday of examination were compared with a *t*-test for the variables with a normal distribution [shown as means (SD)] and with the Kruskal-Wallis test for skewed variables [shown with medians (Q1,Q3)]. Linear regression analysis was used to study the associations of day of the week with concentrations at 0 (plasma glucose, plasma insulin, serum triglycerides) and 120 minutes (glucose and insulin) as well as with early (rAUC_0-30min_) and total relative responses (rAUC_0-120min_) of glucose and insulin. Model 1 was a crude model only including day of the week; Model 2 was adjusted for age, sex, and type 2 diabetes (known type 2 diabetes vs no type 2 diabetes/screen-detected type 2 diabetes); Model 3 was additionally adjusted for body mass index, smoking status, education level, and the Dutch Healthy Eating Index (16); and Model 4 was additionally for variation in time in bed (weekend-weekday), moderate-to-vigorous physical activity (weekend-weekday), and retirement status.

In Model 4, we tested for interactions between weekday and (1) known type 2 diabetes, (2) sex, and (3) retirement status. The test for interaction with known type 2 diabetes was performed because the Maastricht Study has been enriched with individuals with type 2 diabetes. The group without known type 2 diabetes consists of individuals without type 2 diabetes and those with screen-detected type 2 diabetes. An interaction analysis with sex was performed because it has become increasingly apparent that sex differences exist in markers of glucose regulation and their associations with diverse outcomes (18, 19). The rationale for testing for interaction between retirement status and day of the week is that we hypothesize that being retired might even out the differences between weekdays and weekends. For the outcomes with statistically significant interactions (*P*-value for interaction < 0.05), stratified analyses are shown.

The outcome variables were, if needed, logarithmically transformed before analysis to obtain normally distributed model residuals. Accordingly, the variables fasting glucose, 120-minute glucose, all insulin, and triglyceride outcomes were transformed with the natural logarithm.

We performed a number of sensitivity analyses. First, the 4 models were applied to a data set where Saturdays were excluded, as measurements on Saturdays may partly be influenced by the start of the weekend (Friday afternoon/evening behavior). Second, type 2 diabetes status was exchanged with the participants’ hemoglobin A1c (HbA1c) levels. Third, we performed all analyses with the participants that had information on all variables included in Model 4 for each of the outcomes, as there is a relative large amount of individuals with 1 or more missing variables needed in Model 4. The results of the sensitivity analyses are shown in the supplementary material Table S1-S6 (20).

The statistical analyses were performed in R version 3.6.2 (21). A 2-sided ɑ-level of 0.05 was used.

## Results

### Population Characteristics

The characteristics of the study population stratified by day of the week are shown in Table 1. Participants examined on Mondays compared to the other days of the week were similar in relation to age, sex, and other clinical parameters (Table 1). Those examined on Mondays had higher fasting insulin concentrations compared to other days of the week. For both groups, high- and low-density lipoprotein were around 1.5 and 3.0 mmol/L, respectively (Table 2). Boxplots of the 7 outcomes by day of the week are shown in Supplementary Figure 1 (20).

**Table 1.** Characteristics of the study population by weekday of examination

	n	All participants (n = 7635)	Monday (n = 1443)	Tuesday-Saturday (n = 6192)
Sex, men	7635	50.4	50.2	50.5
Age, years	7635	59.8 (8.7)	60.1 (8.9)	59.8 (8.6)
BMI, kg/m^2^	7632	27 (4.5)	27 (4.6)	27 (4.5)
Waist circumference, cm	7630	95.3 (13.7)	95 (13.4)	95.4 (13.8)
Highest education, low/medium/high	7522	(34.6/27.6/37.7)	(37.3/26.9/35.9)	(34.0/27.8/38.2)
Smoking status, never/former/current	7572	(37.2/49.4/13.4)	(38.4/48.2/13.4)	(37.0/49.6/13.4)
Alcohol use,^a^ none/low/high	7571	(18.5/58.2/23.3)	(17.8/61.2/21.0)	(18.7/57.5/23.8)
Physical activity assessed by accelerometer, minutes/day	6412	118.2 (40.9)	118.2 (40)	118.2 (41.2)
Total self-reported physical activity from CHAMPS questionnaire, hours/week	6745	14.0 (8.2)	14.1 (8.5)	13.9 (8.1)
Adherence to Dutch Healthy Diet Index	7144	83.8 (15.1)	84.3 (15)	83.6 (15.1)
Total energy intake, kcal/day	7144	2141.7 (601.8)	2140.7 (617.6)	2142 (598.2)
Glucose tolerance status	7635			
Normal		60.3	61.3	60.1
Impaired fasting glucose		3.6	2.7	3.8
Impaired glucose tolerance		11.4	11.7	11.3
Known type 2 diabetes		20.5	19.9	20.7
Newly diagnosed type 2 diabetes		4.2	4.4	4.2
Duration of type 2 diabetes,^b^ years	1479	6.4 (7.3)	6.4 (7.4)	6.4 (7.3)
Systolic blood pressure	7632	133.7 (17.9)	134.4 (18)	133.6 (17.9)
Diastolic blood pressure	7631	75.6 (9.8)	75.3 (9.8)	75.6 (9.8)
Family history of diabetes	7155	33.4	32.1	33.7
Family history of CVD	7529	28.8	30.5	28.4
Medication, hypertension	7629	38.1	38.7	38.0
Medication, lipid	7629	32.4	32.8	32.3
Glucose-lowering medication	7629	18.4	17.6	18.6
Retired	7077	30.7	33.2	30.1
Difference in time in bed between weekend and weekday, minutes	6203	20.9 (73.3)	23.0 (72.3)	20.4 (73.5)
Difference in MVPA between weekend-weekday, minutes	6128	−3.0 (28.2)	−2.4 (29.4)	−3.2 (27.9)

Data are shown as means (SD) or percentage of population.

Abbreviation: CVD, cardiovascular disease; MVPA, moderate-to-vigorous physical activity.

^a^Alcohol use: low: women: ≤7 units/week; men: ≤14 units per week; high: women >7 units/week; men >14 units/week.

^b^Only those with type 2 diabetes.

**Table 2.** Glucose, insulin, and lipid measurements stratified by weekday of examination

	Monday (n = 1443)	Tuesday-Saturday (n = 6192)	*P*
HbA1c, %	5.8 (0.9)	5.8 (0.9)	0.943
HbA1c, mmol/mol	39.7 (9.4)	39.7 (9.5)	0.971
Fasting plasma glucose, mmol/L	6.0 (1.7)	5.9 (1.5)	0.135
120-minute plasma glucose, mmol/L	7.6 (4.0)	7.6 (4.1)	0.512
rAUC_0-30_ glucose	1.3 (0.1)	1.3 (0.1)	0.495
rAUC_0-120_ glucose	1.4 (0.3)	1.5 (0.3)	0.439
Fasting plasma insulin, pmol/L	64.3 (44.1, 101.4)	60.5 (41.8, 92.3)	0.012
120-minute plasma insulin, pmol/L	359.1 (229.0, 585.3)	329.5 (191.1, 588.0)	0.055
rAUC_0-30_ insulin	3.2 (2.2, 4.6)	3.4 (2.4, 4.9)	0.016
rAUC_0-120_ insulin	5.8 (4.3, 7.6)	6.0 (4.5, 8.2)	0.065
Serum triglycerides, mmol/L	1.4 (0.8)	1.4 (0.9)	0.208
Serum total cholesterol, mmol/L	5.2 (1.1)	5.2 (1.1)	0.081
Serum HDL cholesterol, mmol/L	1.5 (0.5)	1.5 (0.5)	0.607
Serum LDL cholesterol, mmol/L	3.0 (1.0)	3.1 (1.0)	0.005

Differences in characteristics between weekday of examination were compared with a *t*-test for the variables with a normal distribution [shown as mean (SD)] and with the Kruskal-Wallis test for skewed variables [shown as median (Q1,Q3)].

Abbreviations: HbA1c, hemoglobin A1c; HDL, high-density lipoprotein; LDL, low-density lipoprotein; rAUC, relative area under the receiver operating curve.

### Associations of Weekday with Glucose, Insulin, and Triglycerides in the Full Population

The results of the linear regression analyses with estimates of the mean difference (95% CI) between Mondays and the other days of the week for plasma glucose, insulin, and triglyceride concentrations for the full population are shown in Table 3. The glucose measures showed no statistically significant differences between Mondays and the other days of the week in any of the 4 models, except for fasting glucose, which was found to be 1% higher on Mondays in Model 2 to 4.

**Table 3.** Estimates of the mean difference between Mondays vs Tuesday-Saturday (reference group = Tuesday-Saturday) for glucose, insulin, and triglyceride for the entire population

	Model 1		Model 2		Model 3		Model 4	
	n	Estimate (95% CI)	n	Estimate (95% CI)	n	Estimate (95% CI)	n	Estimate (95% CI)
Plasma glucose								
Fasting,^a^ %	7635	1 (0 to 2)	7634	**1 (0 to 2)**	7031	**1 (0 to 2)**	5354	**1 (0 to 2)**
120 minutes,^a^ %	7183	−1 (−3 to 2)	7182	0 (−2 to 2)	6644	0 (−2 to 2)	5095	0 (−2 to 2)
rAUC_0-30_	2944	0.00 (−0.02 to 0.01)	2944	−0.01 (−0.02 to 0.01)	2707	0.00 (−0.02 to 0.01)	1860	0.00 (−0.02 to 0.01)
rAUC_0-120_	2804	−0.01 (−0.05 to 0.02)	2804	−0.02 (−0.05 to 0.00)	2578	−0.02 (−0.05 to 0.00)	1761	−0.02 (−0.06 to 0.01)
Plasma insulin								
Fasting,^a^ %	3360	**10 (2 to 18)**	3360	**9 (1 to 17)**	3078	**9 (3 to 17)**	2107	**9 (1 to 18)**
120 minutes,^a^ %	3062	8 (−1 to 17)	3062	6 (−2 to 15)	2823	5 (−3 to 14)	1949	7 (−3 to 17)
rAUC_0-30_,^a^ %	2927	**−6 (−11 to −1)**	2927	**−5 (−10 to 0)**	2694	−5 (−9 to 0)	1853	−4 (−9 to 2)
rAUC_0-120,_^a^ %	2701	−4 (−9 to 1)	2701	−3 (−8 to 2)	2486	−3 (−7 to 3)	1716	0 (−6 to 6)
Triglyceride								
Fasting,^a^ %	7631	**3 (0 to 6)**	7630	**3 (0 to 6)**	7028	**4 (1 to 6)**	5351	**5 (2 to 8)**

Model 1: crude model only including day of the week. Model 2: Model 1 with additional adjustment for age, sex, and type 2 diabetes status (known type 2 diabetes vs no type 2 diabetes/screen-detected type 2 diabetes). Model 3: Model 2 with additional adjustment for body mass index, smoking status, education level, and the Dutch Healthy Eating Index. Model 4: Model 3 with additional adjustments for variation in time in bed (weekend-weekday), moderate-to-vigorous physical activity (weekend-weekday), and retirement status. Estimates and 95% CIs in bold are statistically significant (*P* < 0.05).

^a^Log-transformed before analysis and back-transformed for presentation.

Fasting plasma insulin and triglyceride concentrations were respectively 9% to 10% and 3% to 5% higher on Mondays compared to the other days of the week in all models in the full population, whereas post-OGTT glucose and insulin concentrations were not statistically significant different between Mondays and the other days of the week in any of the models in the overall population (Table 3).

### Interaction Analyses

We found statistically significant interactions between sex and day of the week for 120-minute glucose and fasting insulin (Table 4). When stratified by sex, there was still no clear differences between Mondays and the other days of the week for 120-minute glucose. However, the association of weekday with fasting insulin concentrations was only apparent for men, where the concentration was 17% to 18% higher on Mondays compared to the other days.

**Table 4.** Estimates of the mean difference between Mondays vs Tuesday-Saturday (reference group = Tuesday-Saturday) for glucose and insulin and triglyceride for the outcomes with a statistically significant interaction between day of the week and either sex or type 2 diabetes status

	Model 1		Model 2		Model 3		Model 4	
	n	Estimate (95% CI)	n	Estimate (95% CI)	n	Estimate (95% CI)	n	Estimate (95% CI)
Stratified by sex								
120-minute glucose,^a^ %								
Women	3642	−2 (−5 to 1)	3641	−1 (−4 to 1)	3384	−2 (−4 to 1)	2620	−2 (−5 to 0)
Men	3541	1 (−3 to 5)	3541	2 (−1 to 5)	3260	2 (−1 to 4)	2475	3 (0 to 6)
Fasting insulin, %^a^								
Women	1622	1 (−7 to 10)	1622	1 (−7 to 9)	1506	2 (−5 to 10)	1042	1 (−8 to 10)
Men	1738	**18 (6 to 33)**	1738	**17 (5 to 31)**	1572	**18 (6 to 30)**	1065	**18 (3 to 35)**
Stratified by T2D status								
Fasting glucose,^a^ %								
T2D	1568	**4 (1 to 7)**	1568	**4 (1 to 7)**	1412	**3 (0 to 6)**	1023	4 (0 to 7)
No known T2D	6066	0 (0 to 1)	6066	0 (0 to 1)	5619	0 (0 to 1)	4331	0 (0 to 1)
rAUC_0-120_ insulin, ^a^ %								
T2D	524	−10 (−20 to 1)	524	−10 (−19 to 1)	477	−9 (−19 to 2)	325	−12 (−25 to 3)
No known T2D	2177	−1 (−7 to 4)	2177	−2 (−7 to 4)	2009	−1 (−6 to 5)	1391	3 (−4 to 10)
Fasting triglyceride,^a^ %								
T2D	1568	4 (−2 to 11)	1568	4 (−2 to 11)	1412	6 (−1 to 13)	1023	**14 (6 to 23)**
No known T2D	6062	3 (0 to 6)	6062	3 (0 to 6)	5616	**3 (0 to 6)**	4328	3 (0 to 6)

Model 1: crude model only including day of the week. Model 2: Model 1 with additional adjustment for age, sex (in the models stratified on T2D status (known T2D vs no T2D/screen-detected T2D), and T2D status (in the models stratified on sex). Model 3: Model 2 with additional adjustment for body mass index, smoking status, education level, and the Dutch Healthy Eating Index. Model 4: Model 3 with additional adjustments for variation in time in bed (weekend-weekday), moderate-to-vigorous physical activity (weekend-weekday) and retirement status. Estimates (95% CIs) in bold are statistically significant (*P* < 0.05).

Abbreviations: rAUC, relative area under the receiver operating curve; T2D, type 2 diabetes.

^a^Log-transformed before analysis and back-transformed for presentation.

In relation to type 2 diabetes, statistically significant interactions with fasting glucose, insulin rAUC_0−120_, and triglyceride were observed, and for all outcomes, the associations were more driven by the group of individuals with known type 2 diabetes (Table 4). In this group, fasting glucose concentrations was 3% to 4% higher on Mondays compared to the other weekdays. For fasting triglycerides, higher concentrations were seen on Mondays compared to the other weekdays for individuals with known type 2 diabetes (Table 4). There were no statistically significant interactions between day of the week and retirement status.

### Sensitivity Analyses

Sensitivity analyses excluding participants examined on Saturdays [Supplementary Tables 1 and 2 (20)], exchanging type 2 diabetes status with HbA1c [Supplementary Tables 3 and 4 (20)], and using a complete-case approach [Supplementary Tables 5 and 6 (20)] were performed. Exclusion of participants examined on Saturdays and exchanging known type 2 diabetes status with HbA1c did not change the results substantially [Supplementary Tables 1-4 (20)]. In the complete-case analysis stratified by known type 2 diabetes status, the estimates for fasting triglyceride changed markedly in Models 1 to 3 so that the estimates became similar for all models [Supplementary Tables 5 and 6 (20)].

## Discussion

In this large Dutch population-based study of men and women with and without type 2 diabetes, we observed 17% to 18% higher fasting insulin concentrations on Mondays compared to the other days of the week in men but not in women. Also, higher concentrations of fasting glucose and triglycerides were observed on Mondays; however, this was mainly the case for a selected group of individuals with known type 2 diabetes. We found no clear differences in glucose and insulin responses during the OGTT between Mondays and the other days of the week.

In a previous study of Danish adults, we found higher fasting insulin concentrations on Mondays compared to the rest of the weekdays combined (10). The observed higher fasting insulin concentrations among men and the trend toward higher triglyceride concentrations on Mondays compared to other weekdays in this study are also in line with a previous study of 581 adolescents, showing a declining trend across the week in concentrations of plasma glucose, insulin, and serum triglycerides (12). Another study in children aged 8 to 11 years found higher insulin resistance and higher concentrations of triglycerides on Mondays compared to Friday (9). Also, a large study using data from blood samples taken as part of routine medical care found higher triglyceride concentrations in the beginning of the week (11). Of interest, they also found that the differences in triglyceride concentrations between weekdays were larger in the younger than in the older population (11). This might explain why we do not see a robust association with fasting triglyceride concentrations in the current study consisting of older adults.

A novel finding of our study was the interaction between sex and weekday on fasting insulin concentrations in adults. Previous studies in adults have not investigated sex differences in the associations reported. However, sex differences in fasting glucose and insulin concentrations in general is a well-known phenomenon (22, 23). In line with this, a study in adolescents found higher variation in insulin across the week in boys than in girls (12). This sex difference may suggest that (1) men have another weekend behavior than women, (2) weekend behavior is acutely affecting metabolism stronger in men than women, or (3) it is a spurious finding caused by, for example, residual confounding. Another interesting finding was that the differences for fasting glucose and triglyceride concentrations were most pronounced in the subpopulation with known type 2 diabetes. This was surprising as we would have expected individuals in treatment for type 2 diabetes to have a more regular weekly behavior due to their medication scheme and increased focus on lifestyle. However, the estimates for fasting glucose were small and not clinically relevant.

The large sample size is a strength of the study. Furthermore, it is a strength that we have been able to adjust for important confounders, although residual confounding is still possible (eg, data on differences in meal timing between week and weekend days were not available). We also performed several sensitivity analyses. First, we found comparable results when individuals examined on a Saturday were excluded, which substantiates the robustness of the findings. In the second sensitivity analyses with exchange of known type 2 diabetes status by HbA1c, the estimates were also comparable to those in the main analyses. The last sensitivity analysis where only individuals who had complete data on all covariates for inclusion in Model 4 were included showed that the triglyceride findings in the stratified analyses for individuals with known type 2 diabetes was influenced by those with more data available. As this study is cross-sectional, we are not able to investigate potential differences in glucose, insulin, and triglyceride concentrations within the same individuals during the week. Also, we have no information on reasons for which day of the week participants had their examination, but we have no reason to believe that the day of examination should be related to any specific factors also related to the outcomes. In the Netherlands, a 4-day work week is not uncommon, and a relatively high proportion of women in particular work part-time (24-26). Therefore, it is not uncommon in the Netherlands to have 1 day off every week, often a Wednesday or a Friday. Unfortunately, we do not have data to account for this in our analyses. We expected a change in estimates between Models 3 and 4 where factors associated with differences between weekends and weekdays were included; however, this was not seen. This could be because the included variables, differences in time spent in bed and physical activity between weekdays and weekends, and retirement status do not capture differences in, for example, the timing of these events.

This study adds knowledge on how potential lifestyle habits during weekdays and on the weekend might influence glucose and lipid metabolism at health examinations. In longitudinal studies or intervention studies, it may therefore be relevant to consider performing health examinations on the same weekday for the same participant when possible to reduce variance in laboratory analysis of blood samples caused by weekday variation. Results from this study can also be used to quantify whether we should be worried about misclassification of glucose tolerance based on fasting glucose samples and OGTTs on different days of the week. Importantly, no weekday differences were seen in the post-OGTT blood samples, and only small differences were observed in fasting plasma glucose concentrations and mainly among people with known type 2 diabetes. These findings suggest that the risk of misclassifying individuals with normal glucose tolerance, impaired fasting glucose, or impaired glucose tolerance due to a weekday effect on glucose concentrations is limited. In the entire population, we found 5% to 9% higher concentrations of fasting insulin and triglycerides on Mondays compared to the other days of the week. It should be noted, however, that a 17% to 18% difference between Mondays and the other weekdays was observed in fasting insulin concentrations among men, which may be of clinical relevance. The differences in fasting glucose and triglycerides were small, and the clinical relevance of these are unclear. However, the results highlight that there seem to be small changes in metabolism during the week, which could be useful for informing interventions aimed at improving metabolic health.

In conclusion, we found 17% to 18% higher fasting insulin concentrations among men on Mondays compared to the rest of the week combined, but with similar fasting glucose concentrations. In a subgroup of people with type 2 diabetes, fasting glucose and triglyceride concentrations were also slightly higher on Mondays compared to the other weekdays; however, these findings were less robust and needs confirmation in other studies.

## Data Availability

Restrictions apply to the availability of some or all data generated or analyzed during this study to preserve patient confidentiality or because they were used under license. The corresponding author will on request detail the restrictions and any conditions under which access to some data may be provided.
